# Identification and Validation of CYBB, CD86, and C3AR1 as the Key Genes Related to Macrophage Infiltration of Gastric Cancer

**DOI:** 10.3389/fmolb.2021.756085

**Published:** 2021-12-07

**Authors:** Haiyan Chen, Qi Sun, Cangang Zhang, Junjun She, Shuai Cao, Meng Cao, Nana Zhang, Ayarick Vivian Adiila, Jinjin Zhong, Chengyun Yao, Yili Wang, Hongping Xia, Linhua Lan

**Affiliations:** ^1^ Institute for Cancer Research, School of Basic Medical Science of Xi’an Jiaotong University, Xi’an, China; ^2^ Department of Pathology, School of Basic Medical Sciences and Sir Run Run Hospital and Key Laboratory of Antibody Technique of National Health Commission, Nanjing Medical University, Nanjing, China; ^3^ Department of Pathogenic Microbiology and Immunology, School of Basic Medical Sciences, Xi’an Jiaotong University, Xi’an, China; ^4^ Department of High Talent and General Surgery and Center for Gut Microbiome Research and Med-X Institute, the First Affiliated Hospital of Xi’an Jiao Tong University, Xi’an, China; ^5^ Department of Orthopedics, Second Affiliated Hospital of Xi’an Jiaotong University, Xi’an, China; ^6^ Jiangsu Cancer Hospital and the Affiliated Cancer Hospital of Nanjing Medical University and Jiangsu Institute of Cancer Research, Nanjing, China; ^7^ Key Laboratory of Diagnosis and Treatment of Severe Hepato-Pancreatic Diseases of Zhejiang Province, the First Affiliated Hospital of Wenzhou Medical University, Wenzhou, China

**Keywords:** macrophage, gastric cancer, WGCNA, immune infiltration, hub genes

## Abstract

Gastric cancer (GC) is rampant around the world. Most of the GC cases are detected in advanced stages with poor prognosis. The identification of marker genes for early diagnosis is of great significance. Studying the tumor environment is helpful to acknowledge the process of tumorigenesis, development, and metastasis. Twenty-two kinds of immune cells were calculated by CIBERSORT from Gene Expression Omnibus (GEO) database. Subsequently, higher infiltration of macrophages M0 was discovered in GC compared with normal tissues. WGCNA was utilized to construct the network and then identify key modules and genes related to macrophages in TCGA. Finally, 18 hub genes were verified. In the PPI bar chart, the top 3 genes were chosen as hub genes involved in most pathways. On the TIMER and THPA websites, it is verified that the expression levels of CYBB, CD86, and C3AR1 genes in tumor tissues were higher than those in normal tissues. These genes may work as biomarkers or targets for accurate diagnosis and treatment of GC in the future. Our findings may be a new strategy for the treatment of GC.

## Introduction

Gastric cancer (GC) has the third mortality rate among cancers worldwide (Siegel et al., 2019). Although the incidence of GC is declining in many countries, its dismal clinical outcome still threatens the health and lives of thousands of people (Siegel et al., 2020). There are disparities between the 5-years survival rates due to various factors, but the survival rates remain very low (Youn et al., 2010; Fields et al., 2011). Besides, it is worth noting that both the morbidity and mortality of GC are still extraordinarily high in China. The majority (95%) of stomach cancers are adenocarcinomas, and no obvious symptoms are observed in the early stage (Zhao et al., 2020; Ma et al., 2016). Surgical resection is the most common treatment for GC, but with a poor prognosis (Liu et al., 2012). In recent years, immunotherapy has been given high expectations. Although immunotherapy has gradually been used as the first or second-line treatment in most kinds of cancers, it was not considered as the preferential treatment of GC. Lacking effective treatment is another problem of advanced GC. Thus, it is urgent to find effective methods for early diagnosis and treatment of GC.

The tumor microenvironment (TME) influences the occurrence and progression of tumors. The complex interaction between tumor cells and tumor-related immune cells occurred in TME (Sperlich et al., 2017). It is mainly composed of tumor-related fibroblasts, immune cells, extracellular matrix, various growth factors, inflammatory factors, special physical and chemical characteristics (such as low oxygen and low pH) and cancer cells. TME plays an essential role in the diagnosis, prognosis, and clinical treatment sensitivity of tumors. The cells in the microenvironment can be grouped into different categories and have complex and significant interactions with each other, and there are some robust cell infiltration patterns (Shaw et al., 2013; Tamborero et al., 2018). It is closely related to clinical prognosis. Thus, the infiltrated immune cells could be used as drug targets to improve the survival rates of cancer patients, which have been recognized in current immunotherapy strategies. By understanding immune cell infiltration, we are capable of decrypting the proportion and functional potential of immune cells in tumor tissues.

With the development of bioinformatics, many algorithms and tools are utilized to explore TME (Lin et al., 2020). Weighted gene co-expression network analysis (WGCNA) is used to find co-expressed gene modules and to explore the association between the gene network and the phenotypes of interest, as well as the core genes in the network (Langfelder and Horvath, 2008). Cell type Identification by Estimating Relative Subsets of RNA Transcripts (CIBERSORT) deconvolutes the expression matrix of immune cell subtypes based on the principle of linear support vector regression. RNA-Seq data can be used to estimate immune cell infiltration in various cancers (Newman et al., 2015). In this study, the WGCNA co-expression network in GC was constructed, and 18 critical genes associated with macrophages were determined. Subsequently, the hub genes and signal pathways were found out in PPI (protein-to-protein interaction). Moreover, we verified that these hub genes were highly expressed in GC at the gene and protein levels. Finally, immunohistochemistry (IHC) results of the collected clinical samples also showed that these genes were highly expressed in GC. In conclusion, CYBB, CD86, and C3AR1 were identified as potential biomarkers in GC.

## Materials and Methods

### Data From the GEO Cohort and Preprocessing

From the Gene Expression Omnibus (GEO) database (http://www.ncbi.nlm.nih.gov/geo/), we downloaded publicly available raw microarray expression data of GSE13911 (https://www.ncbi.nlm.nih.gov/geo/query/acc.cgi?acc=GSE13911) to acquire the transcriptional data of GC. Among the datasets of GC in GEO, GSE13911 has paired normal tissues, containing 31 normal samples and 38 tumor tissues. The chip standardization method was RMA (Robust Multichip Average algorithm), referring to the chip tutorial. The standardization process is mainly divided into three steps: Background correction (removing array auto-fluorescence), Quantile normalization (making all intensity distributions identical), and Probeset summarization (calculating one representative value per probeset).

### Quantification of Immune Tissue Cells

CIBERSORT, a deconvolution algorithm, can calculate the cell composition of complex tissues based on the standardized gene expression data and change the method to energize the abundance of specific cell types. The composition of immune cells in breast and liver cancer tissues was verified and successfully evaluated by flow cytometry (Hackl et al., 2016). It provides 22 kinds of common infiltrating cell expression data LM22 as a reference. Then, we employed the “CIBERSORT” packages in the R to evaluate the infiltration scores of 22 types of immune cells (Langfelder and Horvath, 2008). CIBERSORT was constructed based on microarray data. Thus we applied CIBERSORT for quantitative analysis of immune cells in GEO data.

### Data Preprocessing of GC in TCGA

RNA-seq and corresponding clinical data of GC from the TCGA database were acquired. The RNA-seq data was in the form of HTSeq-FPKM (fragments per kilobase of transcript per million). Thirty-two normal tissues and 381 tumor tissues were screened for further study.

### Co-Expression Network Construction

A weight co-expression network was constructed by using the R package “WGCNA” (12). WGCNA is suitable for complex data analysis in multiple samples. It can calculate the expression connection between genes, identify gene modules with similar expression patterns, analyze the relationship between the gene set and the sample phenotype, and plot the regulation between the genes in the gene set, and appraise key regulatory genes. Through this analysis, we were able to identify the co-expressed gene set, which is called modules. We also associated modules with phenotype data for further investigation and discovered potential marker genes.

The first step was to filter the gene expression data. Missing values or genes with low expression were removed. The samples with many missing values were also deleted. WGCNA has a built-in test gene and sample function, and a basic filter could be performed through this function. After filtering, we proceeded to check if there were samples of outliers and judged the clustering tree of the samples.

When constructing a co-expression network, the correlation coefficients between genes were multiplied to characterize their correlation. The power value was determined by the soft threshold, or what we call the power value and the beta value.

### Differentially Expressed mRNAs Between GC and Non-Tumorous Tissues

“Limma” package was used to screen out the differentially expressed mRNA. FDR-Filter = 0.01 and logFC-filter = 1 were used as the critical values. Differentially expressed genes (DEGs) were screened out for follow-up research.

### Correlation Analysis of Immune Cell Infiltration and Clinical Information

The corresponding clinical information organized in TCGA was analyzed. The clinical information matrix was combined with the immune cell expression matrix. Xcell (Rscript, web tool) (https://xcell.ucsf.edu/) calculated the content of 22 kinds of immune cells to visualize the changes of macrophages in different clinical stages based on ssGSEA (Aran et al., 2017). Through the survival package in R, survival analysis of immune cells and the evaluation of their effects on tumors were attached.

### Functional Enrichment Analysis

With the “clusterProfiler” R package, Gene Ontology (GO) enrichment analysis was realized to explore the molecular functions (MF), biological processes (BP), and cellular components (CC) related to DEGs. From the Kyoto Encyclopedia of Genes and Genomes (KEGG), numerous functions of hub genes were presented.

### Protein-Protein Interaction Network Analysis

STRING is an online tool used to predict the functional interactions between proteins, which is essential for recognizing the mechanisms of cell activities at the molecular levels in cancer progression (Szklarczyk et al., 2019). Using Search Tool for the Retrieval of Interacting Genes (STRING, version 11.5, http://string-db.org) database, a Protein-Protein Interaction (PPI) network was constructed based on 18 genes that positively correlated with macrophage infiltration in GC. The disconnected nodes were hidden in the network, and the PPI network was visualized by the Cytoscape software (version 3.7.2, http://www.cytoscape.org/).

### The Validation in Tumor Immune Estimation Resource

TIMER (https://cistrome.shinyapps.io/timer/) uses RNA-Seq expression profiling data to detect immune cell infiltration in tumor tissues (Li et al., 2017). It provides the infiltration scores of 6 kinds of immune cells (B cells, CD4^+^ T cells, CD8^+^ T cells, Neutrophils, Macrophages, and Dendritic cells). SCNA (somatic copy number alteration) module explores the relationship between somatic cell copy number variation and immune infiltration. TIMER uses GISTIC2.0 data to examine the effect of different gene copy states on immune infiltration compared with normal tissues. The SCNA module is grouped according to the CNA (copy number alteration) of a certain gene. This module explores the different levels of infiltration in 6 kinds of immune cells between the groups. The grouping of CNA is divided into five situations: deep deletion (−2), arm-level deletion (−1), diploid/normal (0), arm-level gain (1) and high amplification (2).

### Gene Expression Level in Gene Expression Profiling Interactive Analysis

GEPIA (http://gepia.cancer-pku.cn/) generates a gene expression boxplot and stages plot based on user-defined input. The specific gene expression of the normal tissues was compared with tumor tissues. Based on the section of the pathological stage, the violin diagram was generated according to the pathological stages of the cases (Zhou et al., 2019).

### The Human Protein Atlas Database

The Human Protein Atlas project (https://www.proteinatlas.org/) contains the protein expression of normal cells, tissues, and cancers. More than 50% of all human protein-encoding genes in line with 11,200 unique proteins were included (Zhou et al., 2018). Through this website, IHC was used to compare the expression of different proteins between human normal tissues and cancer tissues.

### Immunohistochemistry

Paraffin-embedded GC tissues were collected from the First Affiliated Hospital of Xi’an Jiaotong University. Supplementary Table S2 shows the characteristics of patients. IHC was performed by using the EnVision immunohistochemistry kit (Dako, Denmark, k5007). Firstly, the slides were toasted at 60°C overnight. Secondly, the slides were deparaffinized with xylene and rehydrated in gradient ethanol baths. 0.01 M citrate buffer was applied to retrieve the antigen. A 3% H_2_O_2_ solution was dropped in methanol to block endogenous peroxidase. Then, slides were treated with 5% normal goat serum (AR0009) (BOSTER Biological Technology Co. Ltd.) and incubated in the wet box at 37°C for 30 min to block non-specific antigen. Later, commercially available primary antibodies: CD86 (sc-28347), CYBB (sc-130543) (both from Santa Cruz Biotechnology, Inc.), and C3aR (ER 1904-90) (Hangzhou HuaAn Biotechnology Co., Ltd.) were incubated at 4°C overnight (dilution, 1:500). Sections were washed with TBST (Tris Borate Saline Tween-20) 5 min for 3 times. The tablets were incubated with a biotinylated secondary antibody against mouse and rabbit at 37°C for 30 min 3,3′-diaminobenzidine (DAB) was used as the chromogen. The slices were dyed with hematoxylin and mounted after dehydration. Images were digitally captured by ImageView software.

## Results

### Data Processing

**FIGURE 1:**
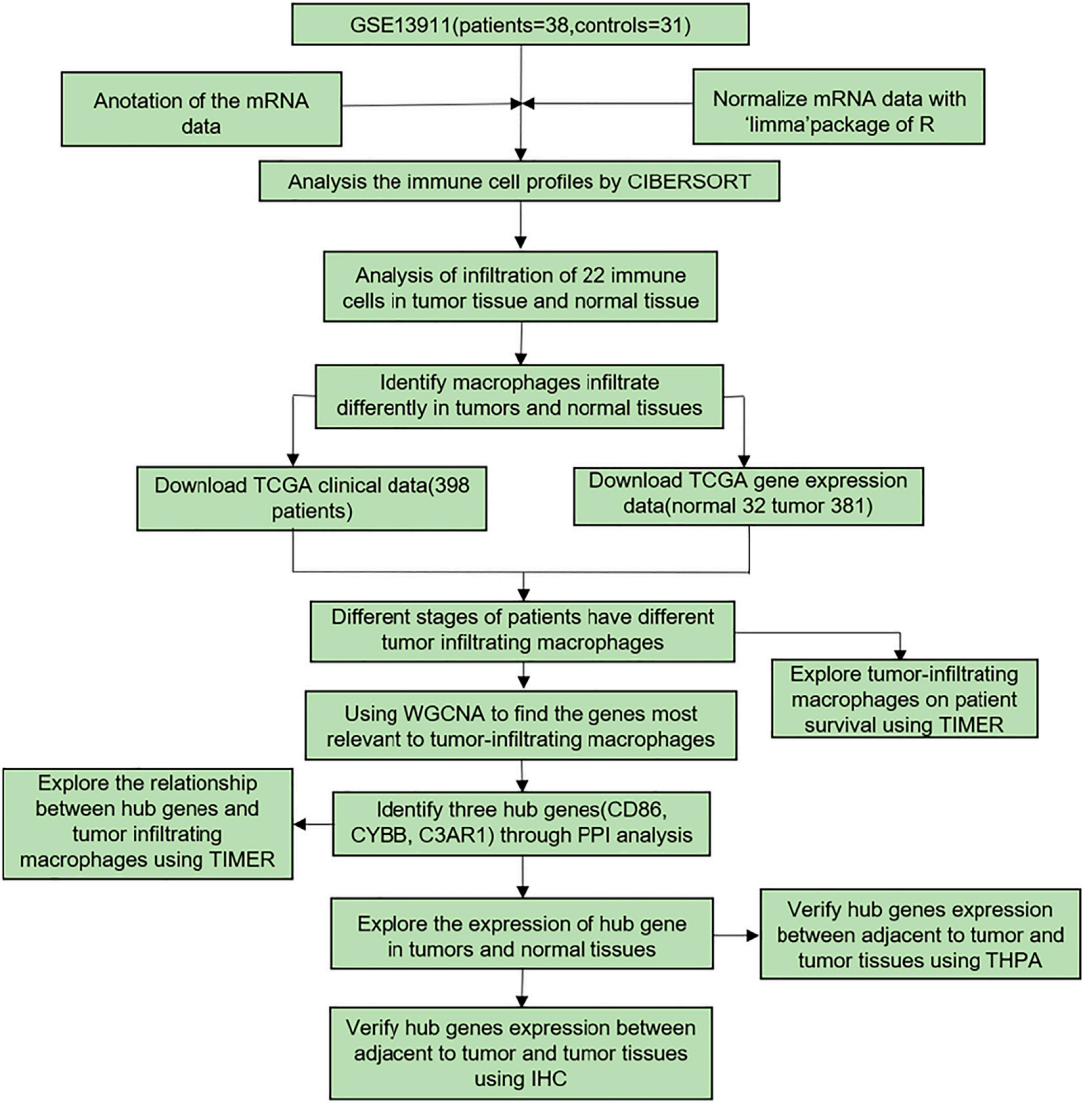
The flowchart of the data processing. GSE13911 was used to analyze the immune cell profiles by CIBERSORT. Corresponding TCGA gene expression and clinical data were used to find the genes related to tumor-infiltrating macrophages by WGCNA. Through PPI analysis, 3 hub genes were identified. The relationship between hub genes and tumor-infiltrating macrophages was explored by TIMER. The expression of hub genes in tumor and normal tissues was identified in GEPIA. HPA was utilized to verify the expression of hub genes between adjacent non-tumorous tumor and tumor tissues. This result was validated by IHC.

As shown in Figure 1, the flowchart presented the process. Firstly, we analyzed the GEO dataset GSE13911. Then, we found that macrophage infiltration was different in GC and normal tissues. To verify this phenomenon, we downloaded related data from TCGA. In this section, we identified three hub genes relevant to macrophages by using WGCNA. Finally, the former results were verified by various websites and IHC.

**FIGURE 2:**
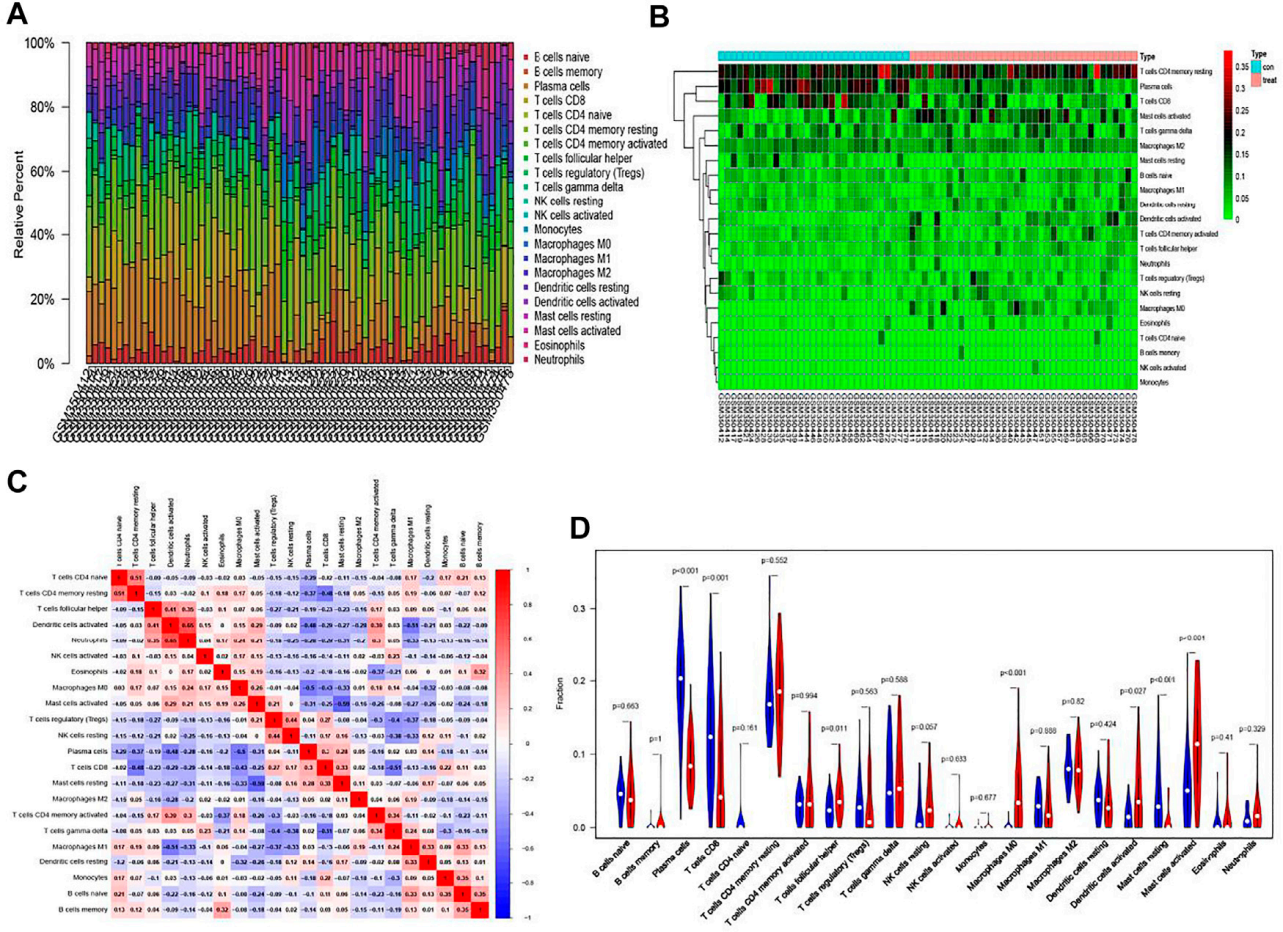
CIBERSORT algorithm for immune infiltration analysis of GEO data. **(A)** Relative percent of 22 kinds of immune cells in GEO. **(B)** A heat map of the cell infiltration data in 69 samples. **(C)** CorHeatmap of 22 immune cells in normal and tumor tissues. **(D)** A comparison of normal and tumor tissues. Blue represent normal controls. Red represent tumor group.

Figure 2A (barplot) showed the ratio of immune cell infiltration in 69 samples. Thirty-one samples on the left are normal tissues, 38 samples are tumor tissues on the right. A heat map (Figure 2B) of the cell infiltration of about 69 samples presented that there was a significant difference in the distribution of some cells in normal and tumor tissues. For example, the proportion of resting memory CD4^+^ T cells was high in the tumor group, while plasma cells and CD8^+^ T cells have a higher proportion in the normal group. CorHeatmap (Figure 2C) was used to observe the co-expression (red) or co-inhibition (blue) of various cells in tumor tissues. We could summarize from the picture that neutrophils and activated dendritic cells had the highest correlation and the correlation coefficient was 0.65. In contrast, resting and activated mast cells were most negatively correlated and the coefficient was −0.59. Then, 31 pairs of samples could be matched in the GSE13911 data set for pairwise comparison. The violin plot was a variation of the box plot, which combines the distribution kernel density estimation curve with the box plot. Follicular helper T cells and M0 macrophages activated dendritic cells, and activated mast cells have a high proportion in the tumor group (Figure 2D). The difference was statistically significant. In the normal group, plasma cells and CD8^+^ T cells and resting mast cells were highly expressed. The most important finding was that the proportion of M0 macrophages in the tumor was significantly higher than the normal group. Besides, the trends of M1 and M2 macrophages were also consistent with the general knowledge in immunology. Moreover, the paired plot result was unanimous in the result of the violin plot but merely looked at a certain cell more directly (Supplementary Figure S1). The M0 macrophages were exclusively selected. In the 31 pairs of matched samples, it could be seen that except for individual cases, most of the M0 macrophage infiltration was higher in the tumor group than in the normal group.

### Immune Infiltration of GC in TCGA

**FIGURE 3:**
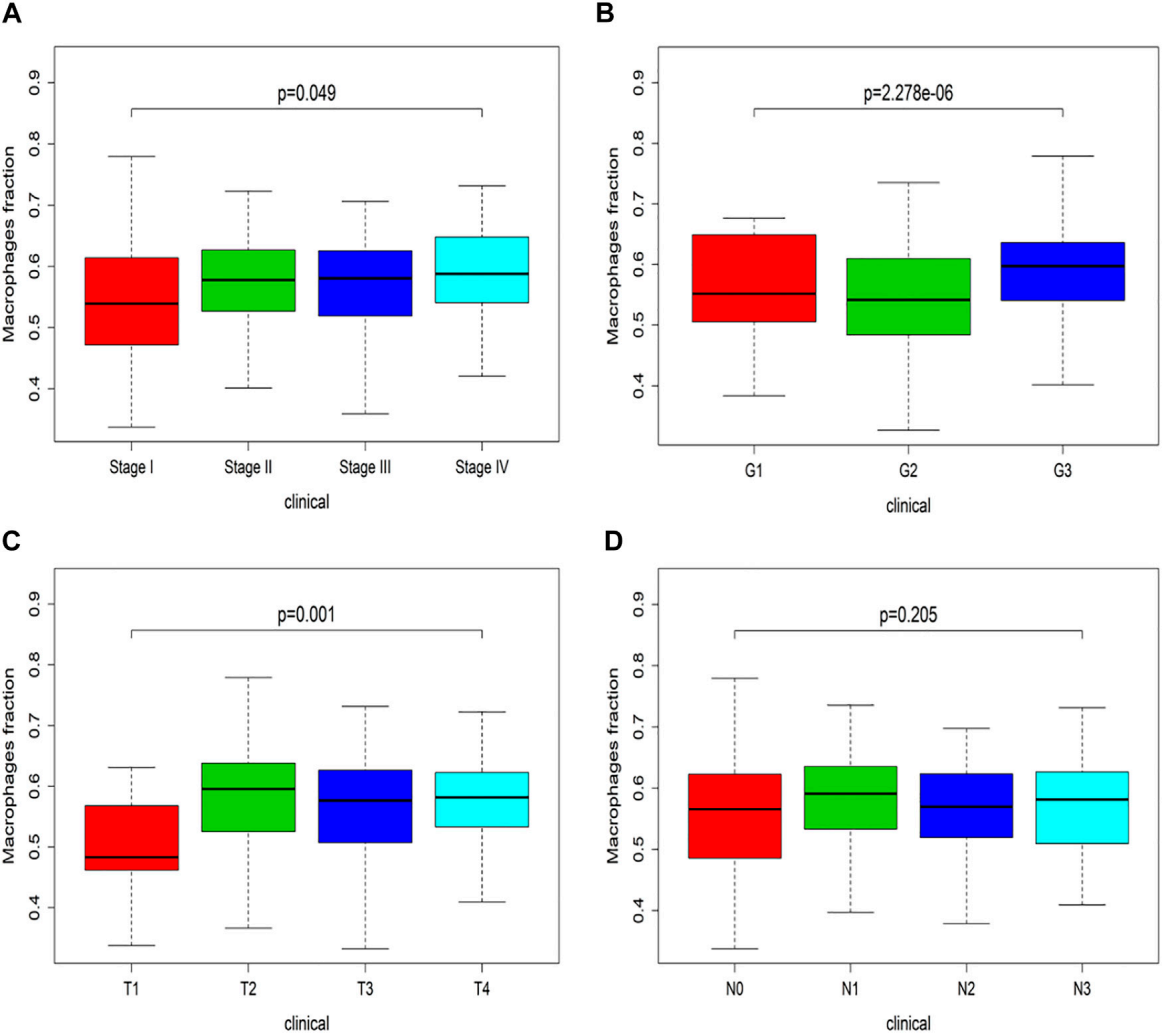
Clinical stage of macrophage in TCGA. **(A)** Macrophage fraction in the I-IV stage. **(B)** Macrophage fraction in G1-G3 grade. **(C)** Macrophage fraction in the T1-T4 stage. **(D)** Macrophage fraction in N0-N3 stage.

Combined with TCGA, the Xcell website was used to analyze the infiltration of immune cells. The changes of macrophages in different clinical stages were observed. In KS multi-group test, the *p*-value indicated the overall situation. The significant discrepancy stated the difference between each group. There was an upward trend in Figure 3, which meant the macrophage fraction was increasing as the clinical-grade increased.

### Differential Analysis of Genes in TCGA Samples

To understand the difference caused by infiltration difference, we used WGCNA to identify the key genes that affect macrophage infiltration. In TCGA, 32 samples were in the normal group and 381 samples were in the tumor group. WilcoxTest was used to analyze the differences between the genes in TCGA samples. A total of 6227 DEGs were picked out.

### WGCNA Co-Expression Network Analysis and Module Mining

WGCNA achieved the goal of quickly locking core genes by grouping genes (modules) and associating gene modules with phenotypes. Next, it was utilized to divide these genes into various modules. Macrophages and neutrophils were used as independent variables to calculate the modules related to macrophages. Finally, Module Membership (MM) > 0.8 and the Gene-Significance (GS) > 0.5 worked as condition to screen out 18 genes that were positively associated with macrophages.

**FIGURE 4:**
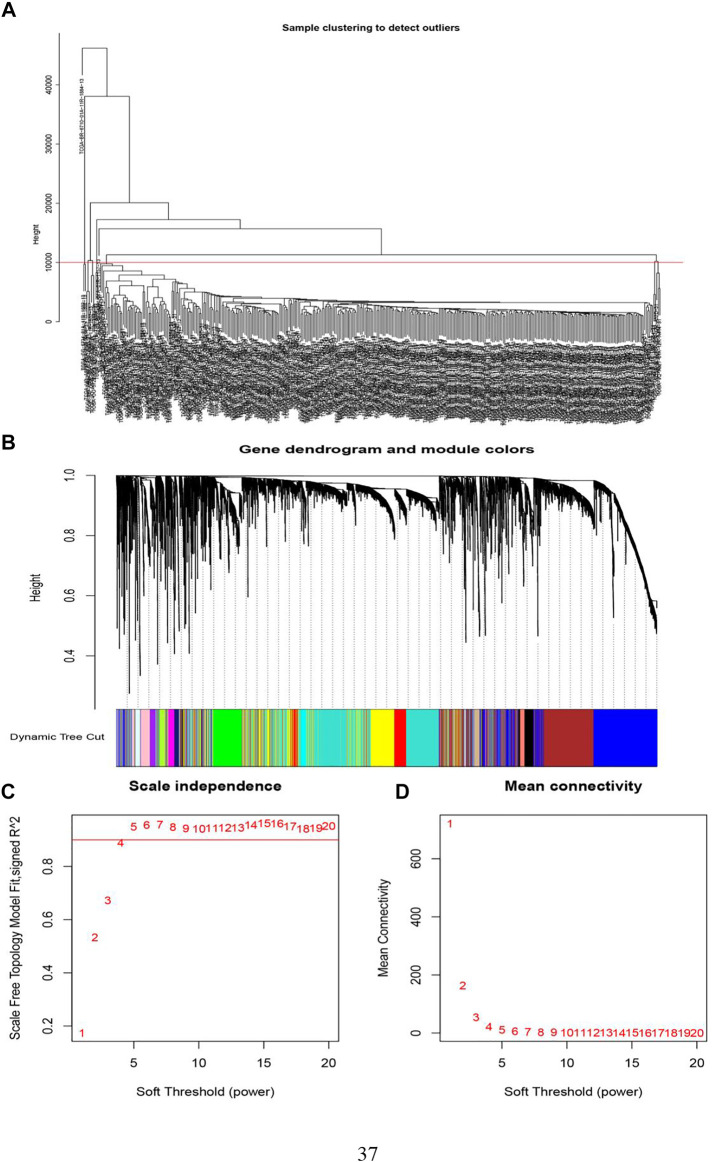
The determination of soft threshold power. **(A)** Screening out the samples with outlier expression. **(B)** By hierarchical clustering, genes were divided into different modules. Different colors equal to different modules. **(C)** Scale-free fit index analysis of 1–20 soft threshold power (*β*). The horizontal axis was the soft threshold (power), and the vertical axis was the evaluation parameter of the scale-free network. The higher the value, the more the network conforms to the non-scale feature (non-scale). **(D)** The average connectivity of soft threshold power was analyzed.

Sample clustering in WGCNA was to screen out the samples with outlier expression and cluster those with a similar expression level. It could be delineated from Figure 4A that the TCGA-BR-6710-01A-11R-1884-13 sample was distinct from other samples. The filtering principle of the soft threshold is to make the network more scale-free. Filtered soft threshold, undirected networks with power less than 15 or directed networks with power less than 30 could not lead to the scale-free network structure *R* ^2^ reaching 0.8 or the average connectivity dropping to below 100. This may result from batch effects, sample heterogeneity, and complicated experimental conditions on expression, which needed to be removed.

WGCNA gene clustering was to cluster genes with similar expression trends. By using a dynamic tree cut, a hierarchical clustering tree was formed. Its rationale was a module (pathway) based analysis (Wang et al., 2019). On the tree, each leaf represented a gene. The genes with similar expression data were tightly connected, formed a branch of the tree, and represented a gene module. After that, several modules were generated (Figure 4B).

The next step was to choose a suitable cutting position. We calculated all integers from 1 to 20 as thresholds to test the optimal threshold. Among them, power Estimate was the best power value, while fit Indices saved the characteristics of the network corresponding to each power and k is the connection degree value. The average value of the connection was visualized and then generated Figures 4C,D. When the *y*-axis was equal to 0.9, the intersection with the curve was exactly equal to 4. Therefore, we chose 4 as the power value.

**FIGURE 5:**
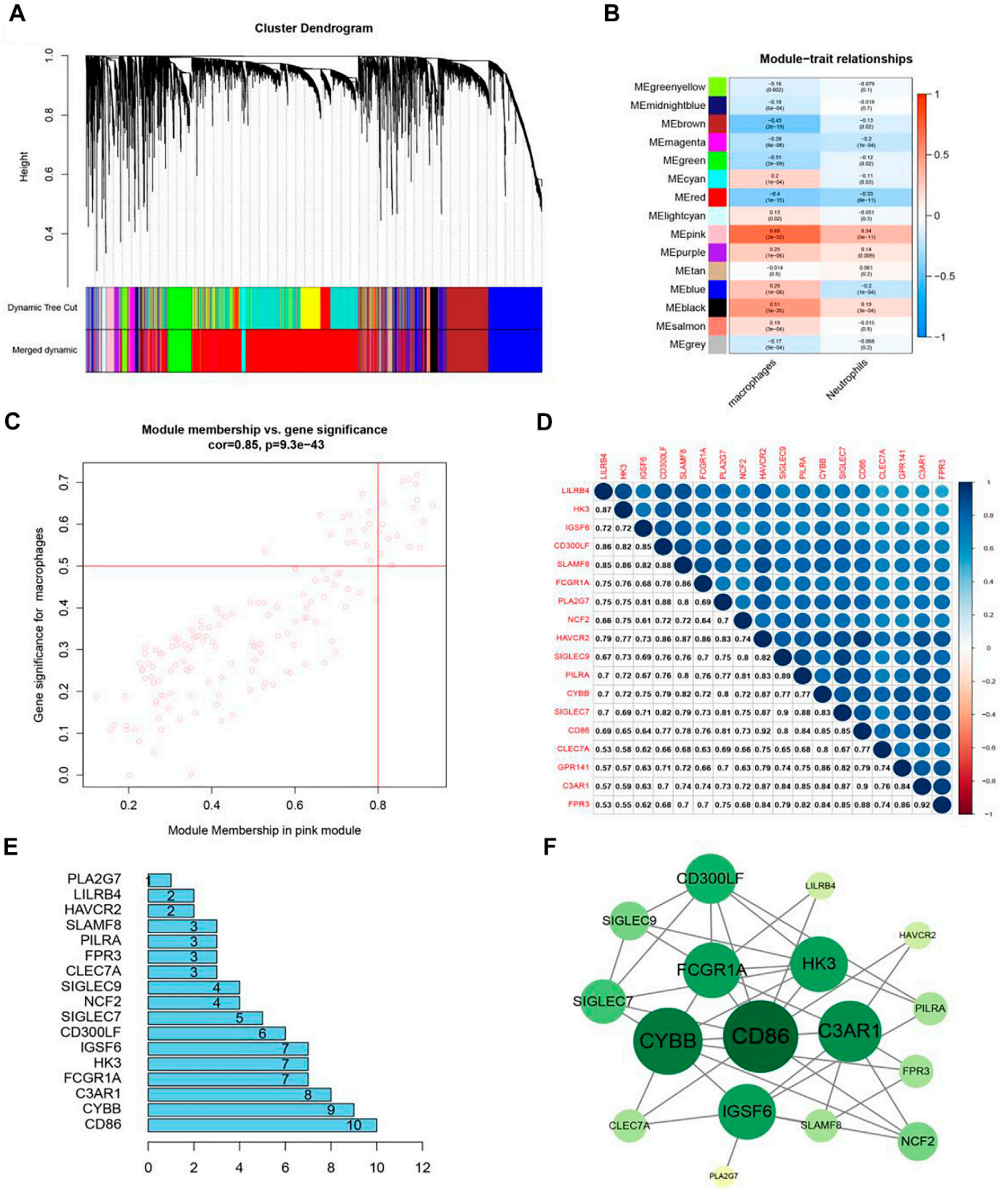
The identification of key modules and genes. **(A)** Co-expression gene modules were acquired from a hierarchical cluster dendrogram. **(B)** Heatmap displays correlations of module eigengenes with macrophages. **(C)** In the MEpink module, each dot represented a gene. The upper right corner of the scatter plot showed genes under the condition of MM > 0.8 and GS > 0.5. **(D)** The correlation heat map of 18 genes. **(E)** Core genes in PPI. **(F)** Co-expression of key genes and upstream genes.

To merge the clustered gene modules and make the modules with similar expressions into a large module (Figure 5A), we used 4 as a beta value to build a gene module. The key step was to associate the genes of different modules with the content of macrophages and pick out the modules that were positively correlated with the content of macrophages. The MEpink module in Figure 5B was selected as most positively correlated with macrophages (correlation coefficient = 0.69, *p* = 2e-52) and the MEbrown module was negatively correlated with macrophages. This research focused on the modules that had a poor survival expectation and the MEpink module was positively correlated with the content of macrophages based on this module.

In Figure 5C, the MEpink module (cor = 0.85, *p* = 9.3e-43) had a strong positive correlation with macrophages. Among them, there were 149 genes in the pink module. In case of MM > 0.8 and GS > 0.5, 18 genes most relevant to macrophage infiltration were selected for following research. Table 1 exhibited the function of 18 genes. A barplot was drawn (Supplementary Figure S2), and these genes were highly expressed in tumors. In the heat map, a total of 18 genes were up-regulated in tumor tissues (Supplementary Figure S3). From the correlation heat map (Figure 5D), we could know the co-expression relation between these genes. All the values were greater than 0.5, indicating that these genes were correlated with each pair, which was also confirmed by WGCNA. After finding the modules associated with the phenotypes, the PPI bar chart (Figure 5E) was obtained. Genes were at the core position, and they could be screened according to different conditions. Cytoscape software was used to form Figure 5F. CD86, CYBB, and C3AR1 were identified as hub genes.

**TABLE 1:** Functional roles of 18 hub genes.

No	Gene symbol	Full name	Function
1	HAVCR2	Hepatitis A virus cellular receptor 2	HAVCR2 is an immune checkpoint and together with other inhibitory receptors including programmed cell death protein 1 and lymphocyte activation gene 3 protein mediates the CD8^+^ T-cell exhaustion
2	SLAMF8	SLAM family member 8	This gene encodes a member of the CD2 family of cell surface proteins involved in lymphocyte activation. These proteins are characterized by Ig domains
3	CD300LF	CD300 molecule like family member f	Members of this family are cell surface glycoproteins with a single IgV-like extracellular domain and are involved in the regulation of immune response
4	CD86	CD86 molecule	Receptor involved in the costimulatory signal essential for T-lymphocyte proliferation and interleukin-2 production, by binding CD28 or CTLA-4
5	SIGLEC7	Sialic acid-binding Ig like lectin 7	Putative adhesion molecule that mediates sialic-acid dependent binding to cells. Preferentially binds to alpha-2,3- and alpha-2,6-linked sialic acid
6	PILRA	Paired immunoglobin like type 2 receptor alpha	PILRA is thought to act as a cellular signaling inhibitory receptor by recruiting cytoplasmic phosphatases like PTPN6/SHP-1 and PTPN11/SHP-2
7	CYBB	Cytochrome b-245 beta chain	It is the terminal component of a respiratory chain that transfers single electrons from cytoplasmic NADPH across the plasma membrane to molecular oxygen on the exterior
8	PLA2G7	Phospholipase A2 group VII	The protein encoded by this gene is a secreted enzyme that catalyzes the degradation of platelet-activating factor to biologically inactive products
9	SIGLEC9	Sialic acid-binding Ig like lectin 9	Putative adhesion molecule that mediates sialic-acid dependent binding to cells
10	C3AR1	Complement C3a receptor 1	Receptor for the chemotactic and inflammatory peptide anaphylatoxin C3a. This receptor stimulates chemotaxis, granule enzyme release, and superoxide anion production
11	FCGR1A	Fc fragment of IgG receptor Ia	This gene encodes a protein that plays an important role in the immune response. This protein is a high-affinity Fc-gamma receptor
12	HK3	Hexokinase 3	This gene encodes hexokinase 3. Similar to hexokinases 1 and 2, this allosteric enzyme is inhibited by its product glucose-6-phosphate
13	LILRB4	Leukocyte immunoglobulin-like receptor B4	This gene is a member of the leukocyte immunoglobulin-like receptor family, which is found in a gene cluster at chromosomal region 19q13.4
14	CLEC7A	C-type lectin domain containing 7A	This gene is closely linked to other CTL/CTLD superfamily members on chromosome 12p13 in the natural killer gene complex region
15	FPR3	Formyl Peptide Receptor 3	This gene includes G protein-coupled receptor activity and N-formyl peptide receptor activity. An important paralog of this gene is FPR2
16	NCF2	Neutrophil cytosolic factor 2	This gene encodes neutrophil cytosolic factor 2, the 67-kilodalton cytosolic subunit of the multi-protein NADPH oxidase complex found in neutrophils
17	IGSF6	Immunoglobulin Superfamily Member 6	This gene related to transmembrane signaling receptor activity
18	GPR141	G protein-coupled receptor 141	GPR141 is a member of the rhodopsin family of G protein-coupled receptors (GPRs)

### Verifying the Tissue Expression Level in THPA and GEPIA Database

We obtained the transcription level of real central genes from THPA. As Supplementary Figure S4A exhibited, the expression level of three hub genes was remarkably high in cancer tissues, which also supported our suppose. CYBB, C3AR1, and CD86 were highly correlated and had high expression levels in GC (Supplementary Figures S4C–E). There was a rising trend in Supplementary Figure S4B, but taking out any of them had little effect on survival (*p* > 0.05), indicating that they might work together.

### Validation of the Protein Expression Level by IHC

**FIGURE 6:**
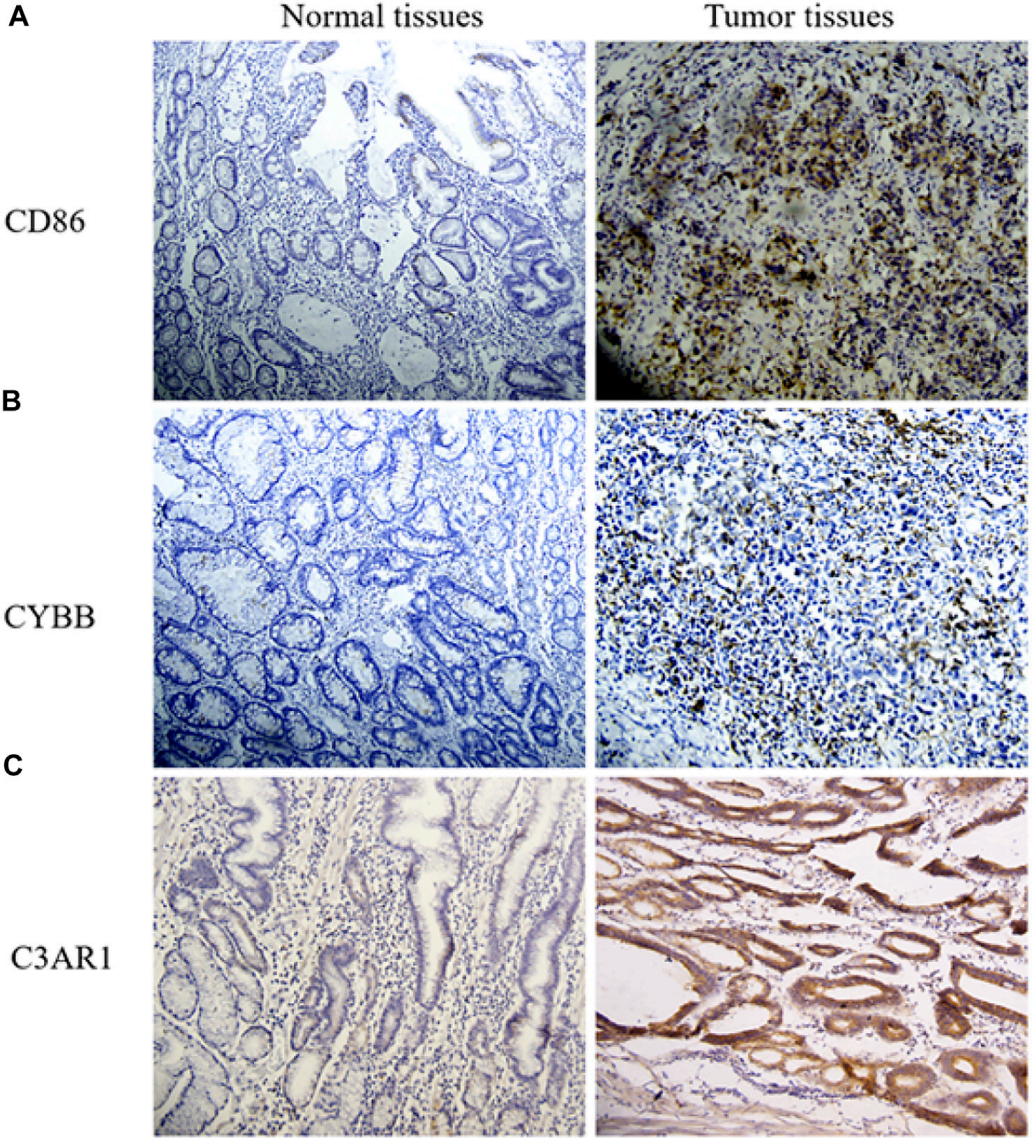
IHC of CD86, CYBB, and C3AR1 in GC and normal tissues. **(A)** The expression level of CD86 in normal and GC tissues. **(B)** Images of CYBB IHC (Original magnification, × 20). **(C)** IHC for C3AR1.

After IHC staining, it was mostly intuitive that the protein level of CD86 was highly up-regulated in GC tissues (Figure 6A). Figure 6B showed a rise in CYBB expression level in tumor tissues compared to adjacent non-tumorous tissues. The positive cell density of C3AR1 was also higher in GC tissues than that in normal tissues (Figure 6C). These results were consistent with THPA. Collectively, these genes regulate the increase of macrophage infiltration in the tumor by affecting several key pathways. In addition, these genes affect monocyte migration.

### Exploring Immune Infiltration by TIMER

**FIGURE 7:**
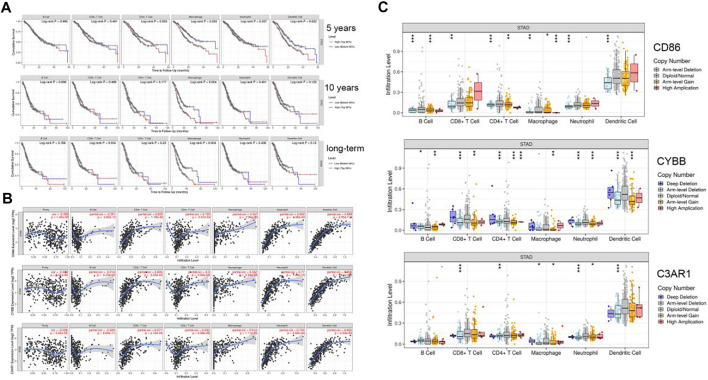
Six kinds of immune cells infiltration situation in TIMER. **(A)** The effect of CD86, CYBB, C3AR1 expression on six kinds of immune cells. **(B)** The survival of immune cells. **(C)** Mutations influence the immune infiltration (*p < 0.05, **p < 0.01, ***p < 0.001).

Using the TIMER website, we found the expression level of CD86, CYBB, and C3AR1 had a positive correlation with CD8^+^T Cells, CD4^+^T Cells, Macrophages, Neutrophils, and Dendritic cells (Figure 7B). The degree of macrophage infiltration significantly affected the prognosis. Hence it is worthy of further research and exploration. These genes affected macrophages and had an impact on survival (5-years survival rate, 10-years survival rate and long-term survival rate) (Figure 7A). To explore the relationship between gene copy number variation and immune infiltration abundance, SCNA module was used to analyze the effect of different somatic CNA of CD86, CYBB, and C3AR1 on the immune cell infiltration in GC. It showed that these genes had a great influence on immune cell infiltration (Figure 7C). These three representative genes identified were associated with tumor-infiltrating macrophages.

### Finding the Biological Pathway Through Gene Function Analysis

**FIGURE 8:**
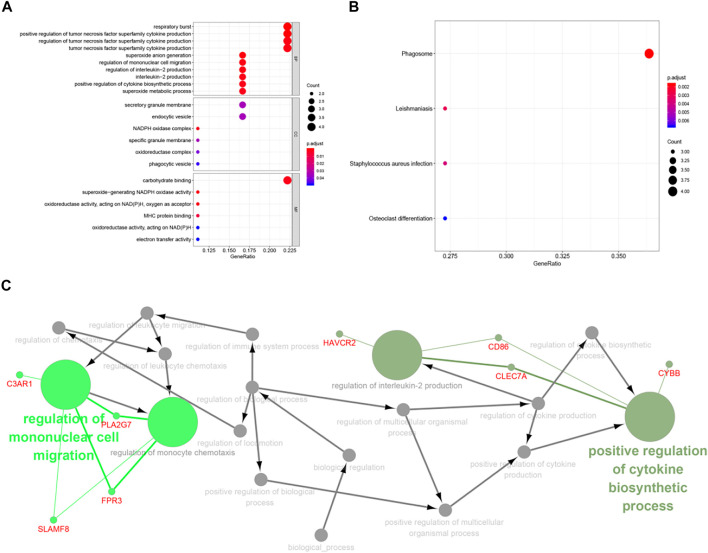
Functional enrichment of key genes. **(A)** The blue module was significantly enriched in GO annotations. **(B)** The blue module was significantly enriched in KEGG pathways. **(C)** Filtering the path with ClueGO.

From the GO enrichment pathway map, we acquainted these 18 genes that were mainly enriched in several pathways (Figure 8A). These genes mainly regulated the production of tumor necrosis superfamily cytokines. Table 2 exhibits the details. Another GO-BP enrichment analysis of DEGs was displayed in Supplementary Table S1. It was delineated that these 18 genes were mainly enriched in the phagosome pathway in the KEGG enrichment pathway map (Figure 8B). GO-CC, GO-MF, and KEGG pathway enrichment analysis of DEGs in GC samples are shown in Table 3.

**TABLE 2:** Top 20 GO-Biological Process enrichment analysis of DEGs in GC samples.

Ontology	Term	Description	Count	p.adjust
BP	GO:0045730	respiratory burst	4	2.42E-05
BP	GO:1903557	positive regulation of tumor necrosis factor superfamily cytokine production	4	4.15E-04
BP	GO:0042554	superoxide anion generation	3	1.09E-03
BP	GO:0071675	regulation of mononuclear cell migration	3	1.60E-03
BP	GO:1903555	regulation of tumor necrosis factor superfamily cytokine production	4	1.60E-03
BP	GO:0071706	tumor necrosis factor superfamily cytokine production	4	1.60E-03
BP	GO:0032663	regulation of interleukin-2 production	3	1.60E-03
BP	GO:0032623	interleukin-2 production	3	2.12E-03
BP	GO:0042108	positive regulation of cytokine biosynthetic process	3	2.39E-03
BP	GO:0006801	superoxide metabolic process	3	2.58E-03
BP	GO:0002479	antigen processing and presentation of exogenous peptide antigen *via* MHC class I, TAP-dependent	3	2.58E-03
BP	GO:0042590	antigen processing and presentation of exogenous peptide antigen *via* MHC class I	3	2.58E-03
BP	GO:0002683	negative regulation of immune system process	5	2.58E-03
BP	GO:0001819	positive regulation of cytokine production	5	2.58E-03
BP	GO:0002430	complement receptor-mediated signaling pathway	2	2.58E-03
BP	GO:0045086	positive regulation of interleukin-2 biosynthetic process	2	2.58E-03
BP	GO:0032760	positive regulation of tumor necrosis factor production	3	2.58E-03
BP	GO:0051249	regulation of lymphocyte activation	5	2.58E-03
BP	GO:0071674	mononuclear cell migration	3	2.73E-03
BP	GO:0002474	antigen processing and presentation of peptide antigen *via* MHC class I	3	3.15E-03

**TABLE 3:** GO-Cellular Components, GO-Molecular Function, and KEGG pathway enrichment analysis of DEGs in GC samples.

Ontology	Term	Description	Count	p.adjust
CC	GO:0043020	NADPH oxidase complex	2	3.69E-03
CC	GO:0030667	secretory granule membrane	3	3.46E-02
CC	GO:0030139	endocytic vesicle	3	3.46E-02
CC	GO:0035579	specific granule membrane	2	3.46E-02
CC	GO:1990204	Oxidoreductase complex	2	4.15E-02
CC	GO:0045335	phagocytic vesicle	2	4.76E-02
MF	GO:0016175	superoxide-generating NADPH oxidase activity	2	2.32E-03
MF	GO:0050664	Oxidoreductase activity, acting on NAD(P)H, oxygen as acceptor	2	2.32E-03
MF	GO:0030246	Carbohydrate-binding	4	2.32E-03
MF	GO:0042287	MHC protein binding	2	9.31E-03
MF	GO:0016651	Oxidoreductase activity, acting on NAD(P)H	2	4.90E-02
MF	GO:0009055	electron transfer activity	2	4.90E-02
KEGG	hsa04145	Phagosome	4	1.65E-03
KEGG	hsa05140	Leishmaniasis	3	2.98E-03
KEGG	hsa05150	*Staphylococcus aureus* infection	3	3.82E-03
KEGG	hsa04380	Osteoclast differentiation	3	6.69E-03

ClueGO, as a Cytoscape plug-in that integrated GO terminology and KEGG/BioCarta pathway was used to create a functionally ordered GO/pathway network (Bindea et al., 2009). After screening (*p* < 0.05), we found C3AR1 participates in the regulation of mononuclear cell migration and monocyte chemotaxis. CD86 partakes in the regulation of cytokine biosynthetic process and interleukin-2 production. CYBB is also involved in the regulation of cytokine biosynthesis (Figure 8C).

## Discussion

Although the incidence of GC continues to decline in Western countries, it is still a common tumor in developing countries. At present, surgery is still the main treatment for localized GC. However, most GC is diagnosed at advanced stages and the recurrence rate remains very high. Combined chemotherapy for patients with GC usually causes drug resistance. In recent years, immunotherapy has become one of the most promising cancer treatment strategies and has had significant effects on several types of tumors (Yang, 2015; Granier et al., 2016; Kerr and Chisholm, 2019). Immunotherapy for GC also has promising prospects (Wu et al., 2019).

Tumor-associated macrophages (TAMs) are one of the most abundant immune cell populations in the TME (Solinas et al., 2009; Franklin and Li, 2016; Pang et al., 2017). TAMs are associated with poor prognosis. While the mechanisms of TAMs in GC are still unclear, further research is urgently needed.

In our study, we calculated the infiltration scores of various immune cells in GC tissues and matched normal tissues (GSE13911) by using CIBERSORT. We found that the M0 macrophage infiltration score in the tumor tissues was significantly higher than the normal tissues (*p* = 0.001). We suspected that tumor progression was related to the degree of macrophage infiltration. To verify our hypothesis, the WGCNA algorithm was performed to find 18 genes that were positively related to the degree of macrophage infiltration in GC. We conducted GO and KEGG enrichment analysis on these 18 genes and found these genes were related to the production of TNF and IL-2 and the formation of phagosomes. Furthermore, we identified three hub genes: CD86, CYBB, and C3AR1, based on the PPI network for further studies. We used TIMER to explore the effect of various immune cell infiltration levels on the survival of GC patients. Moreover, we found that these three genes affected various tumor-infiltrating immune cells, especially tumor-infiltrating macrophages. Again, these hub genes were found to be highly expressed in tumor tissues than normal tissues by using the THPA database. As the tumor progressed, these hub genes expressions were confirmed to rise in GEPIA. Finally, we collected some tumor tissues and adjacent tissue samples in the clinic. Through IHC, we also confirmed that these genes were highly expressed in tumor tissues.

Cytochrome B-245 Beta Chain (CYBB) is expressed in eosinophils, neutrophils (Nauseef and Borregaard, 2014), and B lymphocytes et al., responding to many inflammatory cytokines and stimuli such as IFN-γ, LPS, and TNF-α (Newburger et al., 1991; Frazão et al., 2015). It has been proposed as a primary component of the microbicidal oxidase system of phagocytes (Belambri et al., 2018). CYBB can lead to Immunodeficiency 34 (IMD34) and Granulomatous Disease. The terminal of a respiratory chain needs it. Cellular pH can be regulated by CYBB.

Complement C3a Receptor 1(C3AR1) is activated by its natural ligand, C3a, which is a 77 amino acid split product converted by C3 protein and is traditionally considered to be mainly pro-inflammatory (Coulthard and Woodruff, 2015). After stimulation, chemotaxis, granule enzyme, and superoxide anion were produced (Gaudet et al., 2011). It is widely expressed in a variety of differentiated hematopoietic cell lines in the lung, spleen, ovary, placenta, small intestine, whole brain, heart, and endothelial cells. Also, it was mainly expressed in lymphoid tissue (Brennan et al., 2019).

CD86 is a member of the immunoglobulin superfamily. It works as a ligand for two proteins-CD28 antigen and cytotoxic T-lymphocyte-associated protein 4 (CTLA-4). It is widely known that the CD86 molecule (B7-2) belongs to the B-7 family. Together with the CD80 molecule (B7-1), it is expressed on antigen-presenting cells (APCs, like dendritic cells, macrophages, and B-cells) (Takács et al., 2019). Diseases related to CD86 include acute myocarditis and myocarditis. CD86, as a costimulatory molecule, participates in the formation and regulation of immune synapses (Huemer et al., 2014). Dai et al. showed that CD86 expression in CLL was lower than that in normal B cells, but its role in CLL cell survival was not clear (Dai et al., 2009; Brzostek et al., 2016).

In general, C3AR1 is related to complement function. CYBB and TNF are pro-inflammatory genes. CYBB is related to interaction between neutrophils and macrophages. CD86 is a macrophage activation marker. Its high expression indicates that macrophages are activated. Numerous researches spring up about the identification of prognosis-related genes about GC by WGCNA. For example, one research explored the prognosis of GC but was not related to macrophages (Zhao et al., 2016). Another study aimed to identify potential vital miRNAs and modules associated with the progression of GC (Gong et al., 2019). Our study revealed the important genes and pathways associated with macrophages in GC for the first time.

However, our study had limitations that should be acknowledged. Whether macrophages can convert to each other has always been controversial. The intrinsic response of TAM is the anti-cancer effect of M1 macrophage activation in the process of definite tumor or early carcinogenesis. Subsequently, the tumor cell microenvironment was filled with abundant growth factors and inflammatory mediators [colony stimulating factor-1 (CSF-1), IL-4, IL-10, and TGF-β], which changed the phenotype of macrophages into M2-type macrophages with the function of promoting tumor growth (O’Sullivan et al., 2012). Last but not least, we analyzed the results based on bioinformatics methods and only predicted the relevant results. Therefore, further molecular biological experiments were required to confirm the function of the hub genes associated with GC.

## Conclusion

We identified and verified that the expression levels of CYBB, CD86, and C3AR1 in tumor tissues were higher than those in normal tissues. Together they affect macrophage infiltration. These genes may work as biomarkers or targets for accurate diagnosis and treatment of GC in the future. Our findings may be a new strategy for the treatment of GC.

## Data Availability

The data that support the findings of this study are openly available in TCGA. The data that support the findings of this study are openly available in GEO at https://www.ncbi.nlm.nih.gov/geo/query/acc.cgi?acc=GSE13911.
