# Supplementary figures and images for: Identification and Validation of CYBB, CD86, and C3AR1 as the Key Genes Related to Macrophage Infiltration of Gastric Cancer
Source: Front Mol Biosci. 2021 Dec 7;8:756085. doi: 10.3389/fmolb.2021.756085 (PMC8688826; doi:10.3389/fmolb.2021.756085)

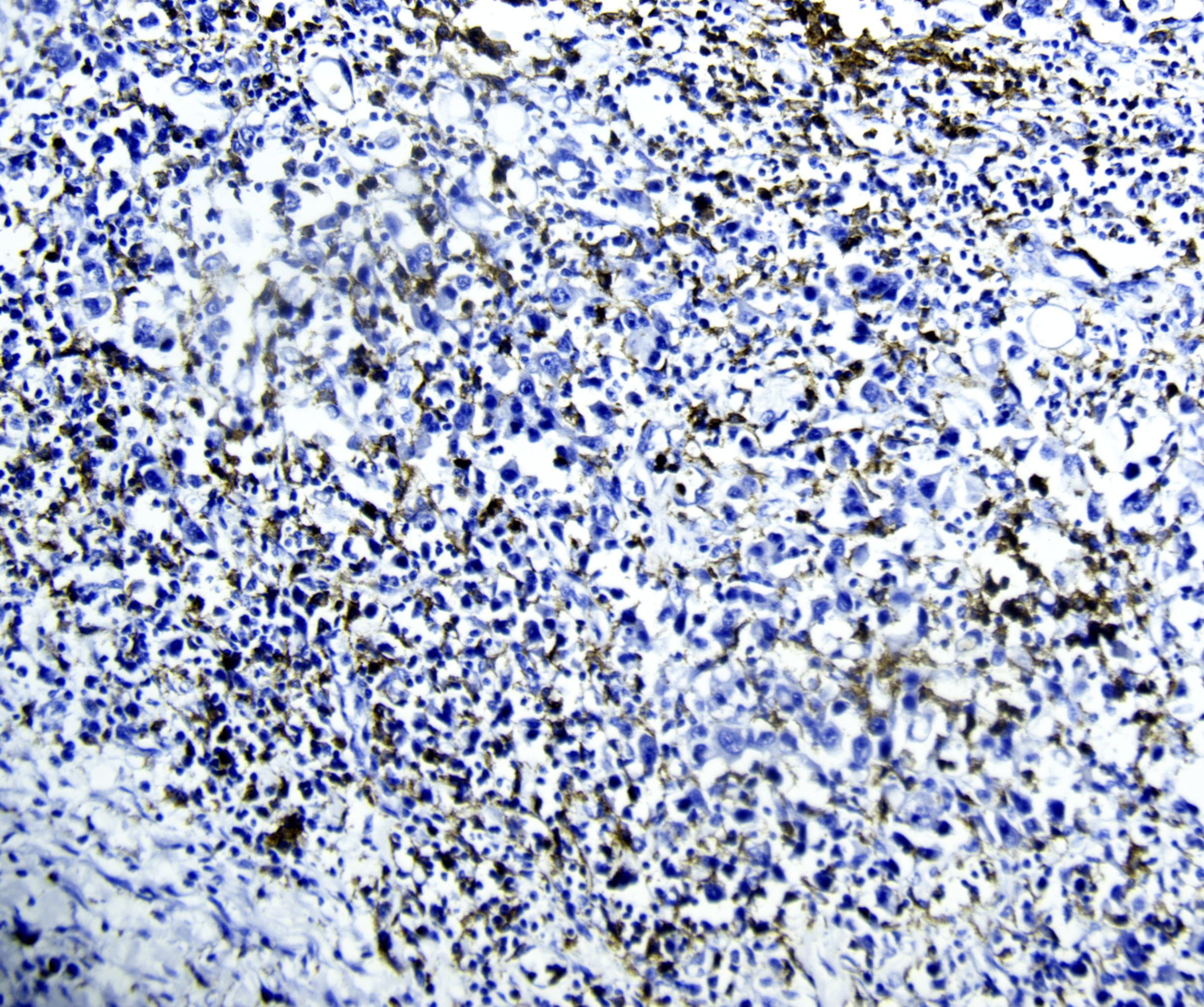

Supplement: Supplementary file 1 [file Image6.TIF]

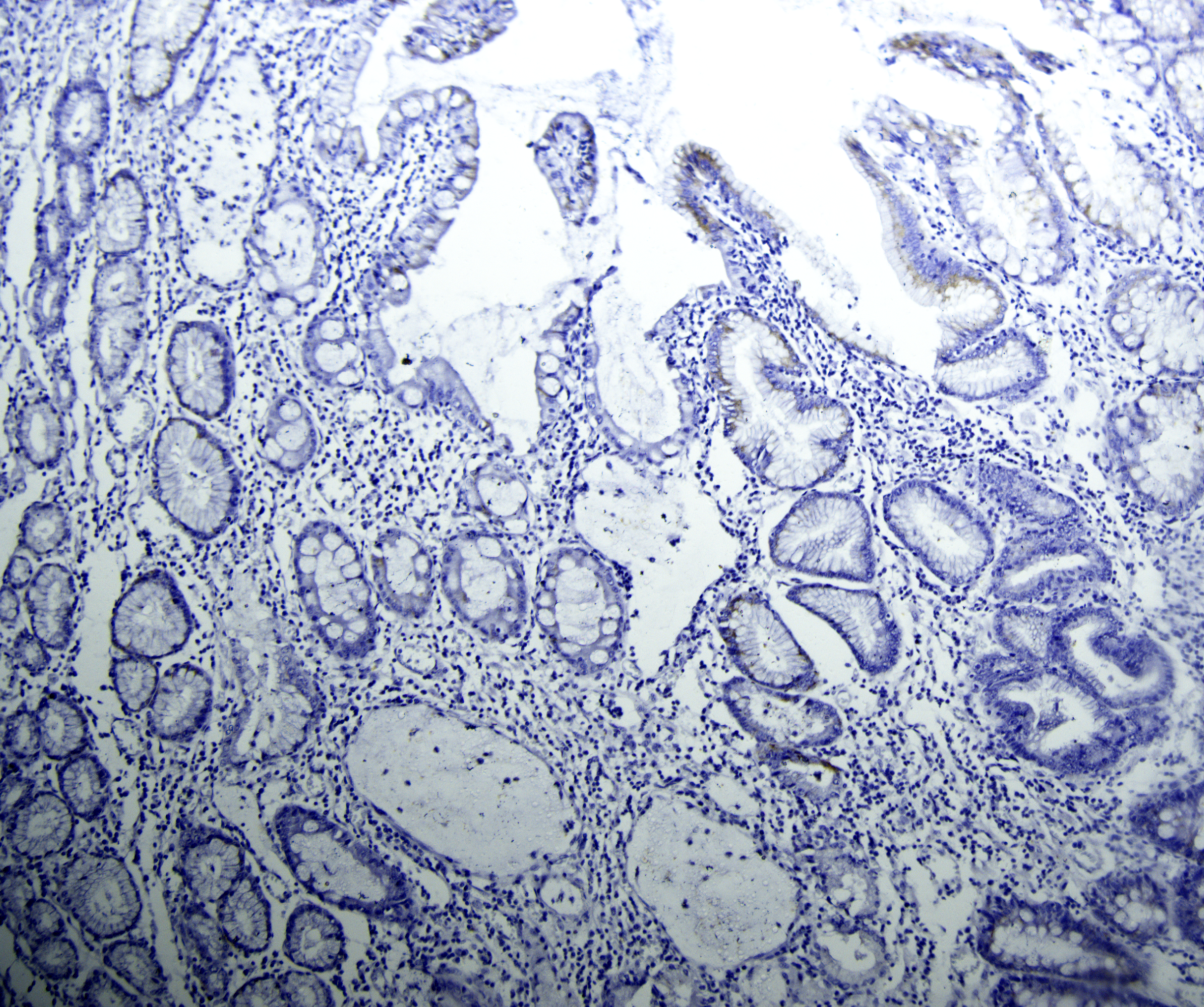

Supplement: Supplementary file 3 [file Image3.TIF]

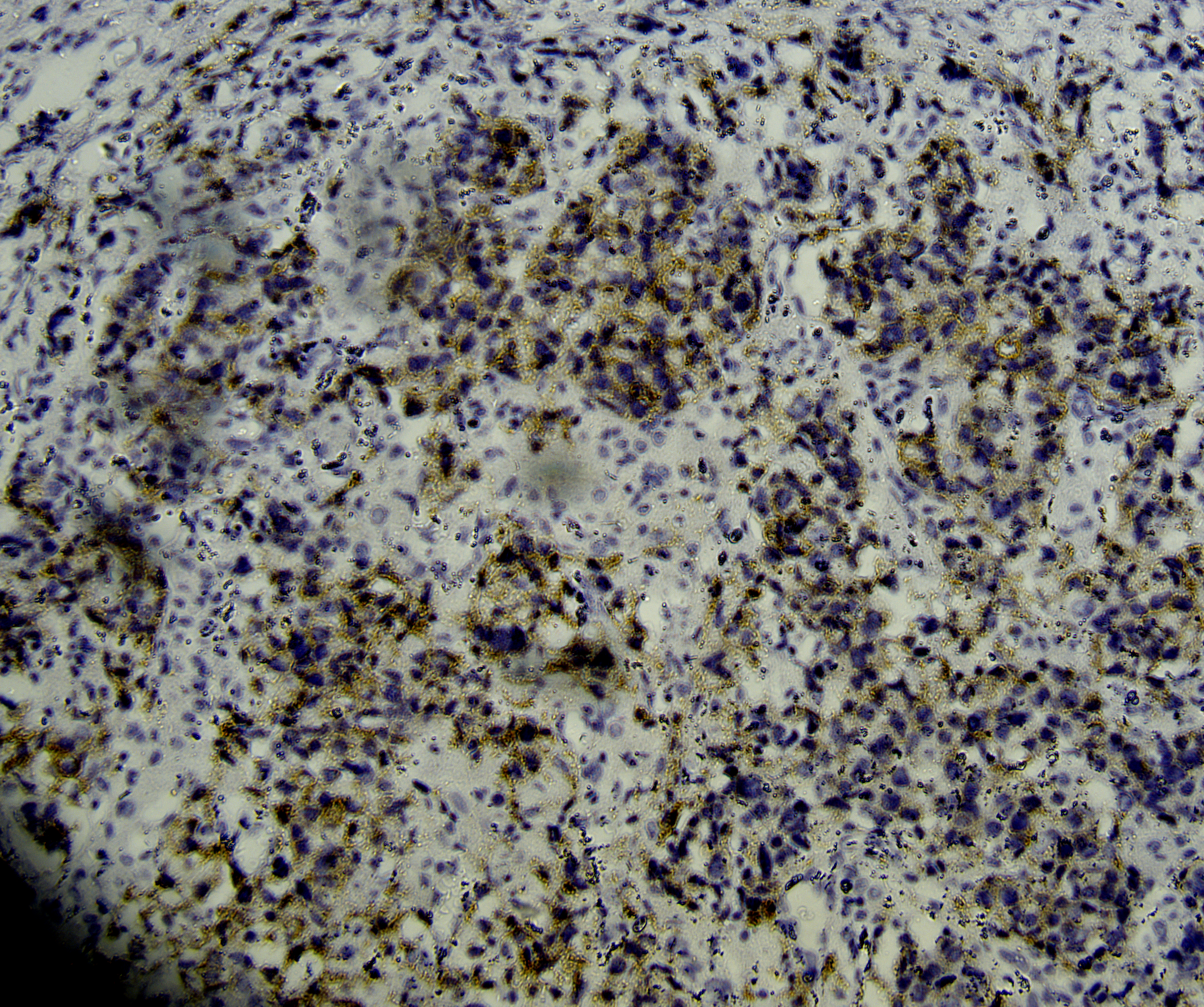

Supplement: Supplementary file 4 [file Image4.TIF]

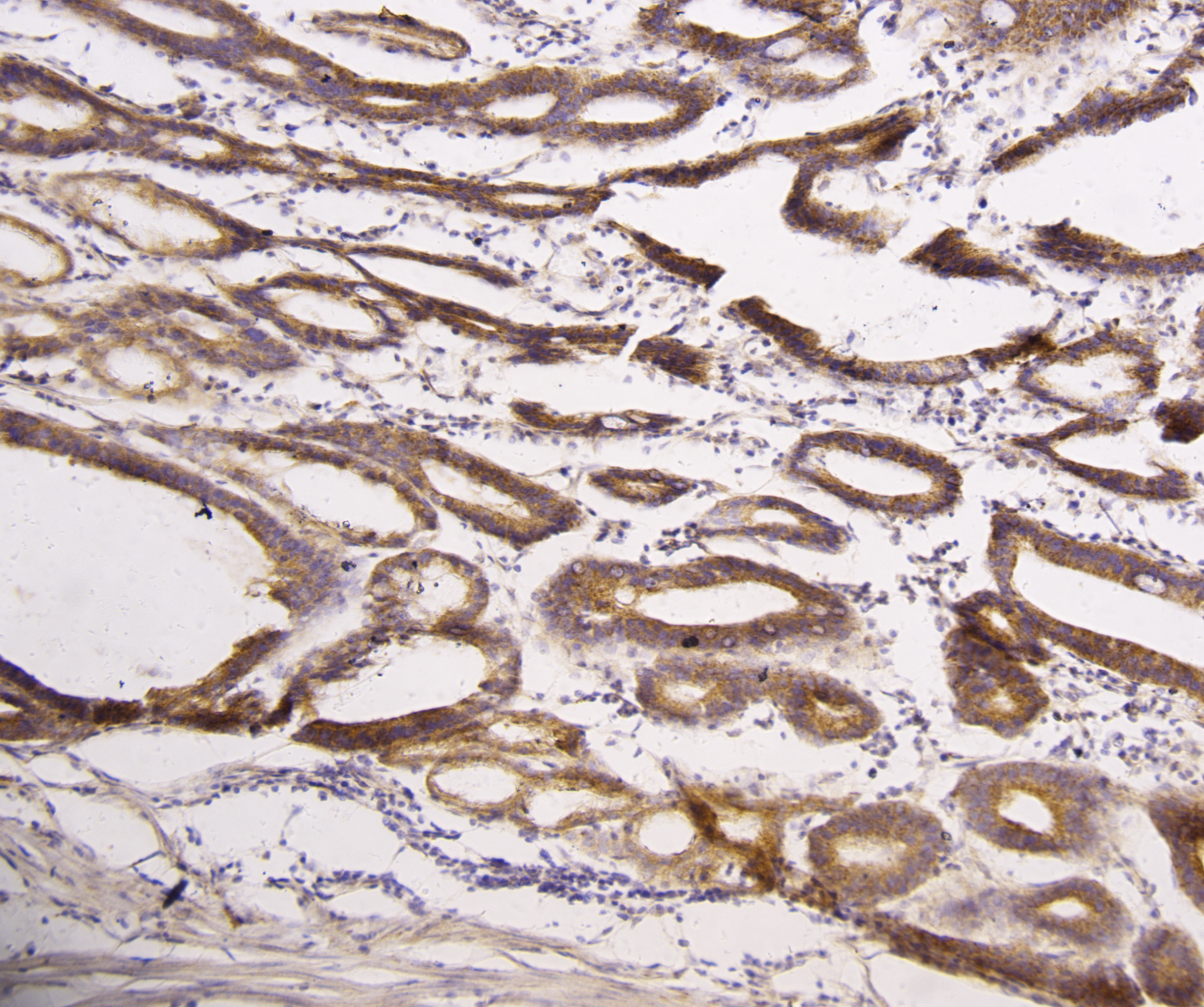

Supplement: Supplementary file 6 [file Image2.TIF]

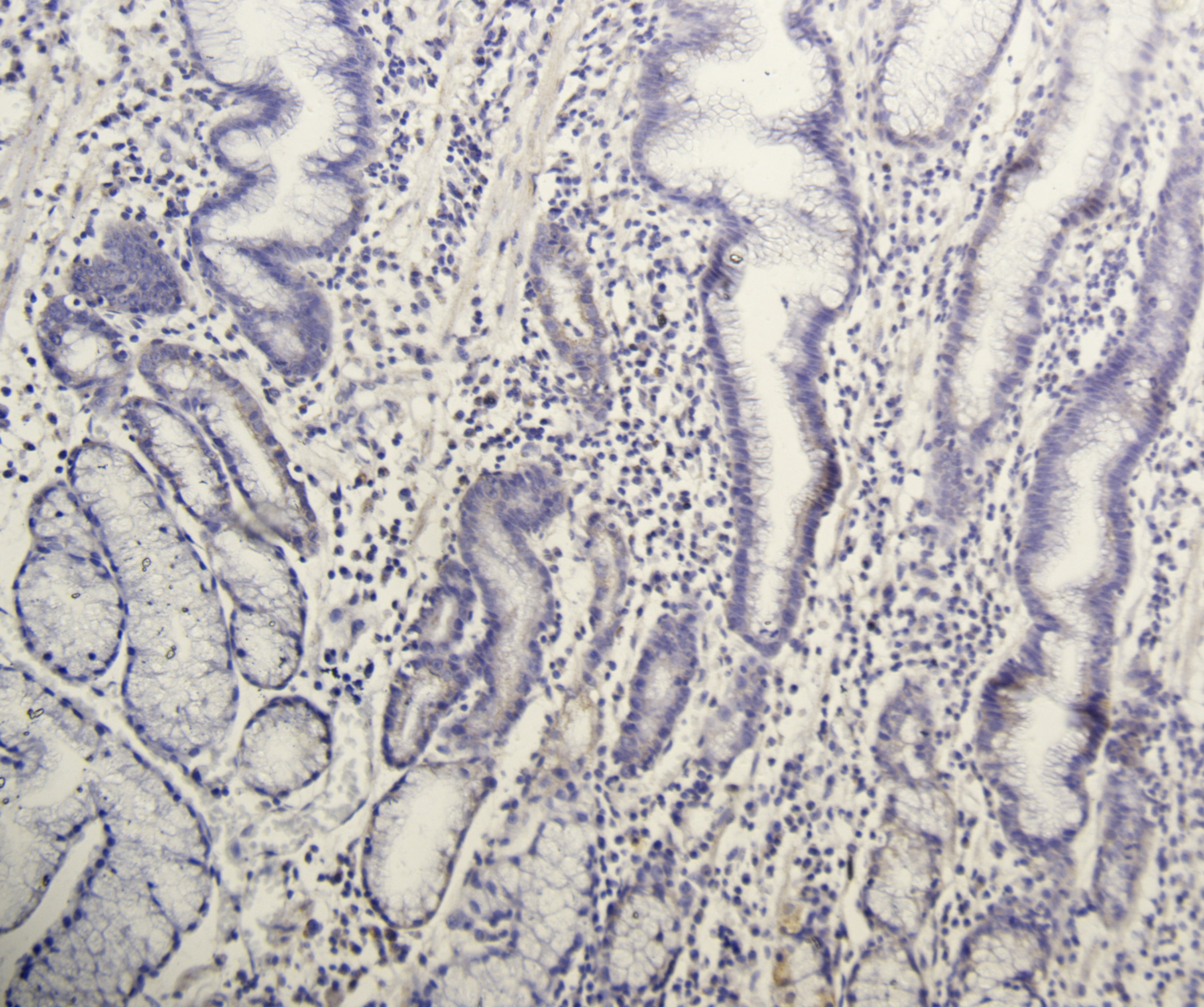

Supplement: Supplementary file 7 [file Image1.TIF]

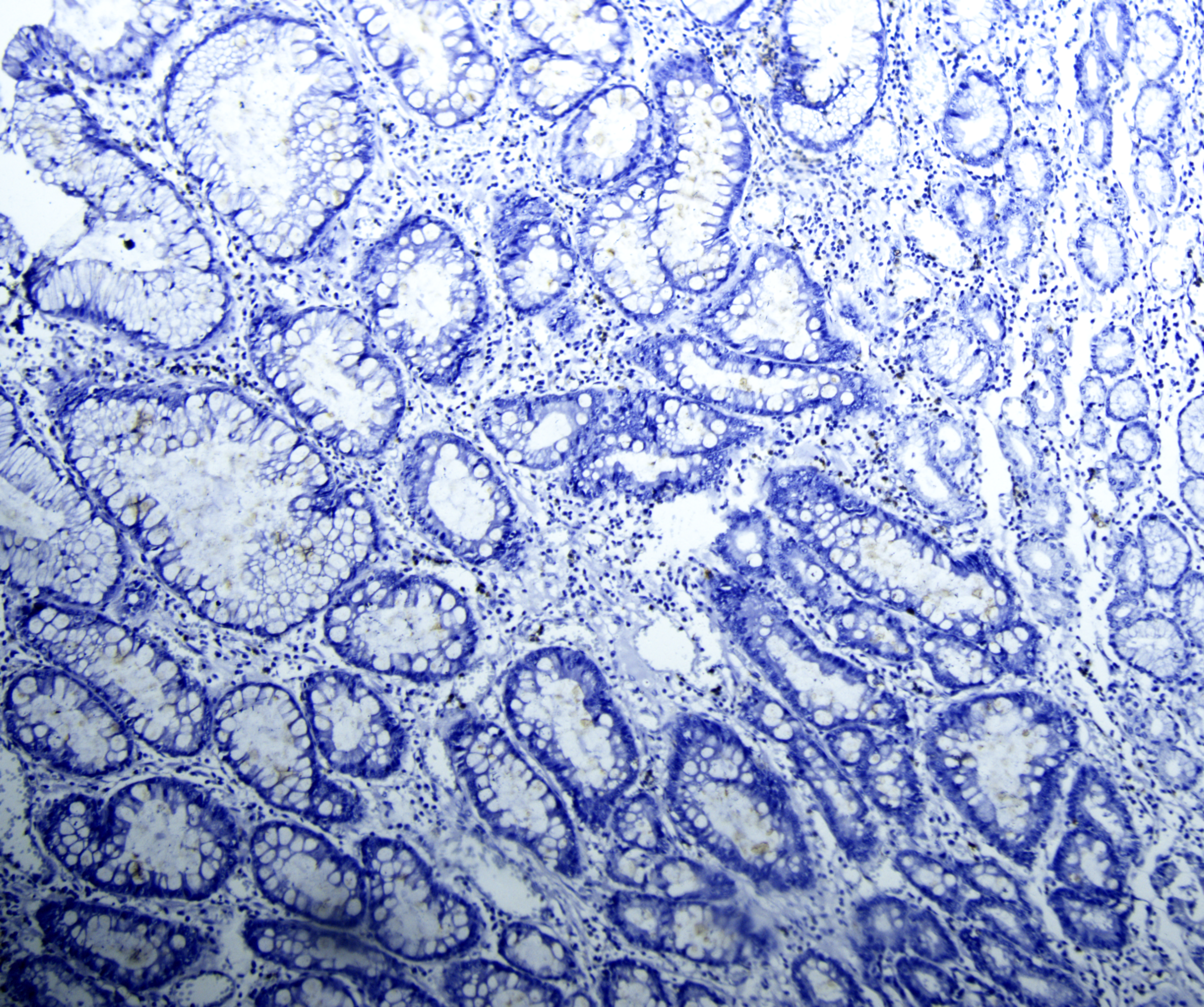

Supplement: Supplementary file 8 [file Image5.TIF]
