# Supplementary material for: Identification and Validation of CYBB, CD86, and C3AR1 as the Key Genes Related to Macrophage Infiltration of Gastric Cancer
Source: Front Mol Biosci. 2021 Dec 7;8:756085. doi: 10.3389/fmolb.2021.756085 (PMC8688826; doi:10.3389/fmolb.2021.756085)
Supplement: Supplementary file 2 [file Presentation1.pdf]

Supplementary Material

Figure S1. The paired plot of the samples in GEO. CD8+T cells and plasma cells, resting Mast cells were lower in tumor group. On the contrary, activated Dendritic cells and follicular helper T cells, macrophages M0 were higher in tumor group. *P<0.05 is considered statistically significant.

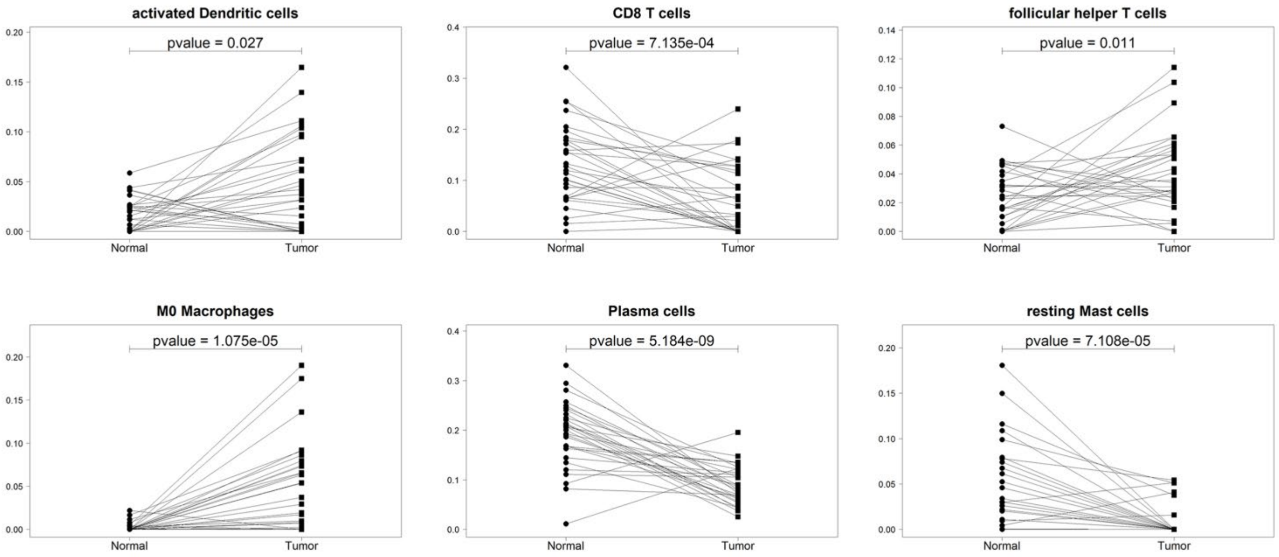

Figure S2. Barplot of the 18 genes. 18 genes were up-regulated in tumor group. All of them were statistically different. Type N represents normal group, type T represents tumor group.

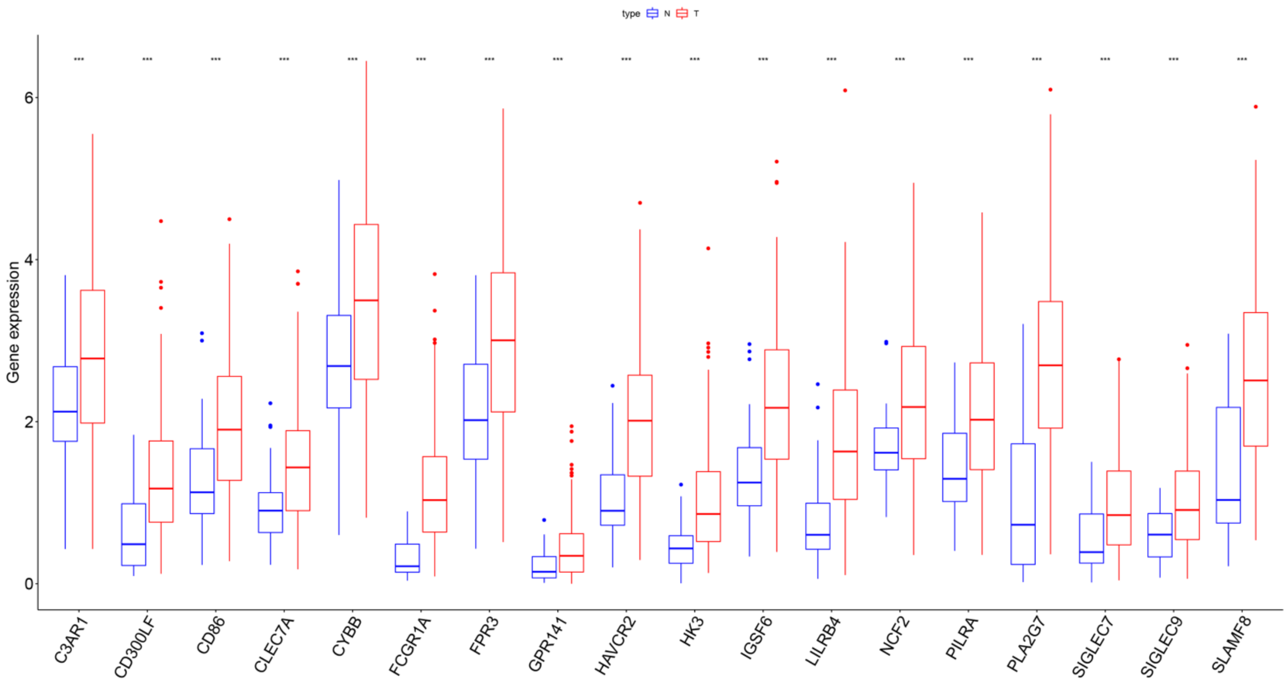

Figure S3. Heat map of 18 genes. The 18 genes were up-regulated in tumor tissues.

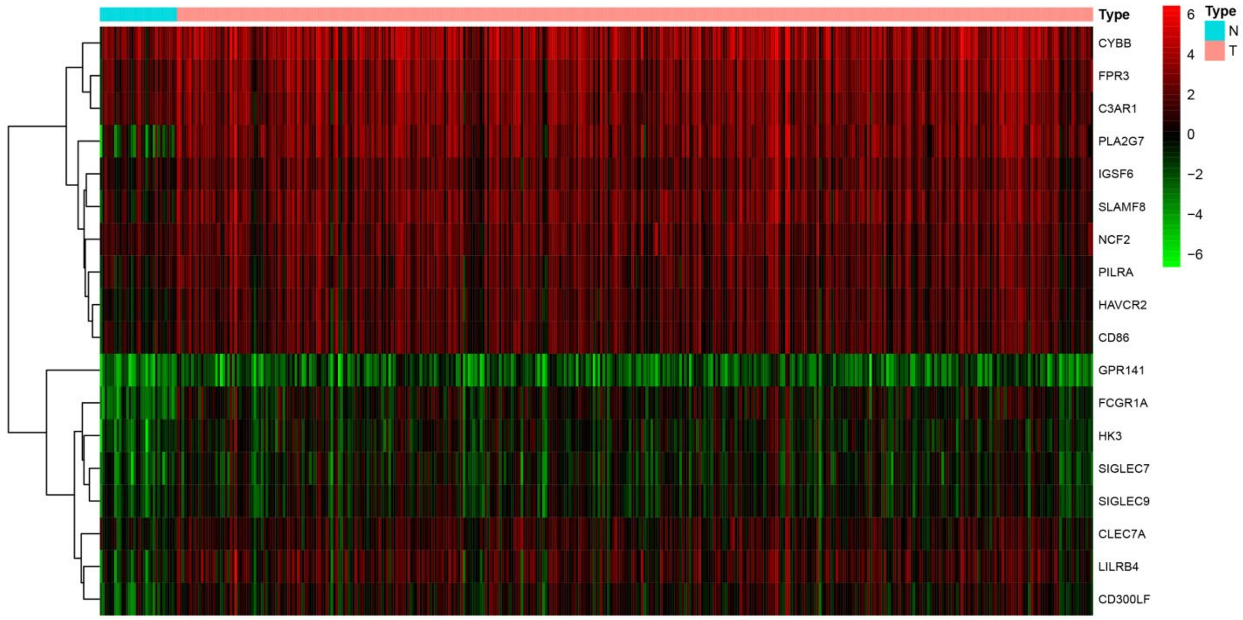

Figure S4. The verification of hub genes on protein level by THPA and GEPIA. (A) In GEPIA the expression level of CD86, CYBB and C3AR1 was showed on the left. Translational level of GC and normal tissues were demonstrated on the upper side and the lower side in THPA. (B) Violin plot of CD86, CYBB and C3AR1 in different stages. (C-E) Co-expression of every two genes.

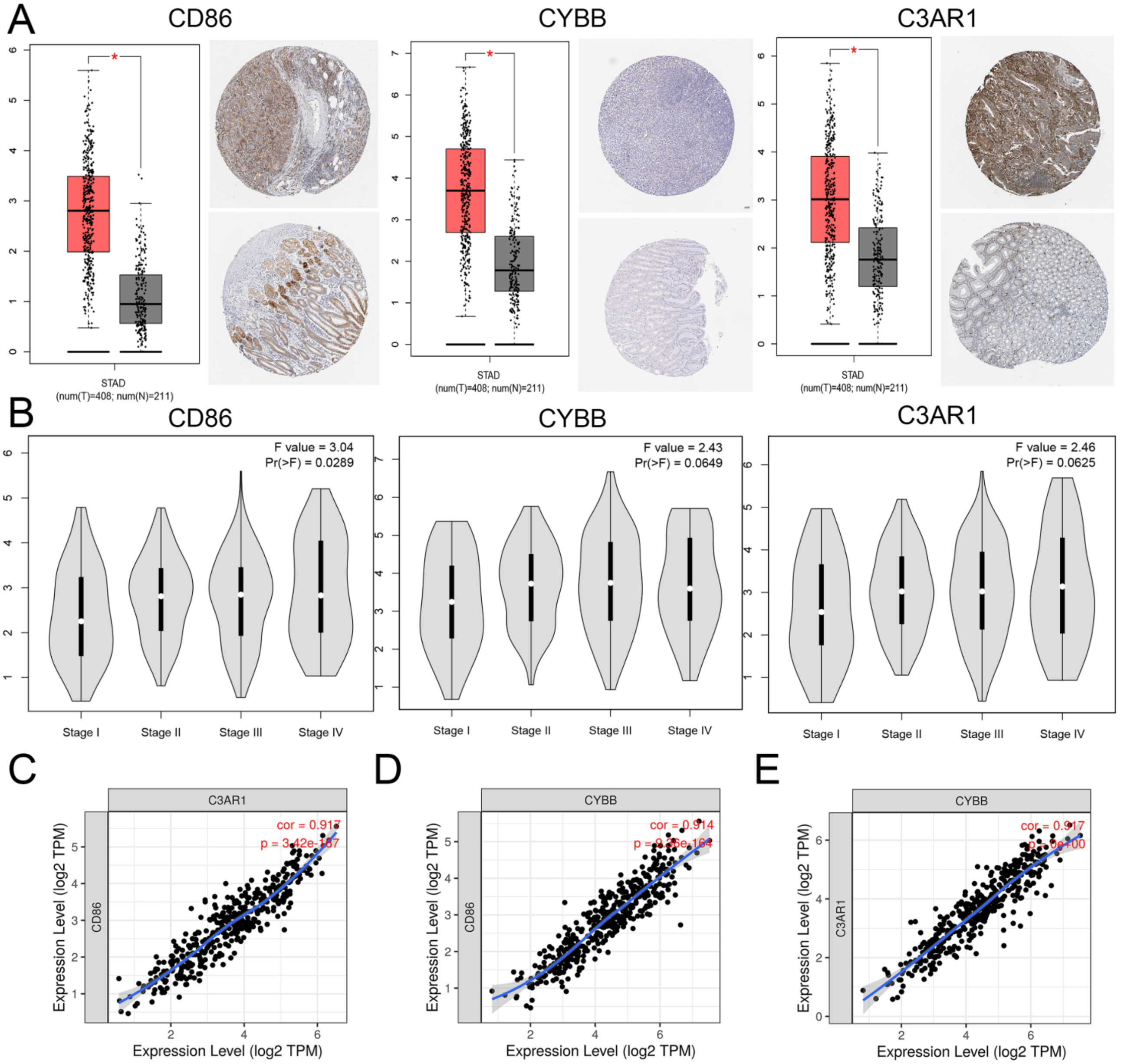

Table S1. Other GO-Biological Process enrichment analysis of DEGs in GC samples.

Ontology	Term	Description	Count	p.adjust
BP	GO:0060263	regulation of respiratory burst	2	3.49E-03
BP	GO:0045076	regulation of interleukin-2 biosynthetic process	2	4.18E-03
BP	GO:0042535	positive regulation of tumor necrosis factor biosynthetic process	2	4.19E-03
BP	GO:0002688	regulation of leukocyte chemotaxis	3	4.19E-03
BP	GO:0042035	regulation of cytokine biosynthetic process	3	4.19E-03
BP	GO:0042094	interleukin-2 biosynthetic process	2	4.58E-03

BP	GO:0042089	cytokine biosynthetic process	3	4.58E-03
BP	GO:0042107	cytokine metabolic process	3	4.58E-03
BP	GO:0032753	positive regulation of interleukin-4 production	2	4.58E-03
BP	GO:0032103	positive regulation of response to external stimulus	4	4.58E-03
BP	GO:0051251	positive regulation of lymphocyte activation	4	5.03E-03
BP	GO:0090025	regulation of monocyte chemotaxis	2	5.03E-03
BP	GO:0010758	regulation of macrophage chemotaxis	2	5.28E-03
BP	GO:0032673	regulation of interleukin-4 production	2	5.53E-03
BP	GO:0006909	phagocytosis	4	6.46E-03
BP	GO:0032743	positive regulation of interleukin-2 production	2	6.46E-03
BP	GO:0002696	positive regulation of leukocyte activation	4	6.90E-03
BP	GO:0032633	interleukin-4 production	2	6.97E-03
BP	GO:0032680	regulation of tumor necrosis factor production	3	6.97E-03
BP	GO:0050867	positive regulation of cell activation	4	6.97E-03
BP	GO:0042533	tumor necrosis factor biosynthetic process	2	6.97E-03
BP	GO:0042534	regulation of tumor necrosis factor biosynthetic process	2	6.97E-03
BP	GO:0032640	tumor necrosis factor production	3	6.97E-03
BP	GO:0045619	regulation of lymphocyte differentiation	3	7.46E-03
BP	GO:1902622	regulation of neutrophil migration	2	7.46E-03
BP	GO:0002478	antigen processing and presentation of exogenous peptide antigen	3	7.80E-03
BP	GO:0002695	negative regulation of leukocyte activation	3	7.80E-03
BP	GO:0048246	macrophage chemotaxis	2	7.80E-03
BP	GO:1905521	regulation of macrophage migration	2	8.05E-03
BP	GO:0019884	antigen processing and presentation of exogenous antigen	3	8.26E-03
BP	GO:0048002	antigen processing and presentation of peptide antigen	3	9.04E-03
BP	GO:0045088	regulation of innate immune response	4	9.43E-03
BP	GO:0002685	regulation of leukocyte migration	3	9.45E-03
BP	GO:0002697	regulation of immune effector process	4	9.45E-03
BP	GO:0002221	pattern recognition receptor signaling pathway	3	9.45E-03
BP	GO:0050866	negative regulation of cell activation	3	9.49E-03
BP	GO:0042110	T cell activation	4	9.49E-03
BP	GO:0050870	positive regulation of T cell activation	3	9.64E-03
BP	GO:0002429	immune response-activating cell surface receptor signaling pathway	4	9.85E-03
BP	GO:0060263	regulation of respiratory burst	2	3.49E-03

$P<0.01$ is considered statistically significant.

Table S2. Characteristics of Patient

Variables	Statistics(N=179)
Age	
N(miss)	179(0)
Mean±SD	59±12
Min-Max	23-86
Median	60

Gender	
Female	36(20.1%)
Male	143(79.9%)
OS Status	
Live	94(52.5%)
Died	85(47.5%)
